# Children and adults minimise activated muscle volume by selecting gait parameters that balance gross mechanical power and work demands

**DOI:** 10.1242/jeb.122135

**Published:** 2015-09

**Authors:** Tatjana Y. Hubel, James R. Usherwood

**Affiliations:** Structure and Motion Laboratory,The Royal Veterinary College, Hatfield, Hertfordshire AL9 7TA, UK

**Keywords:** Walk, Run, Gait, Scaling, Children

## Abstract

Terrestrial locomotion on legs is energetically expensive. Compared with cycling, or with locomotion in swimming or flying animals, walking and running are highly uneconomical. Legged gaits that minimise mechanical work have previously been identified and broadly match walking and running at appropriate speeds. Furthermore, the ‘cost of muscle force’ approaches are effective in relating locomotion kinetics to metabolic cost. However, few accounts have been made for why animals deviate from either work-minimising or muscle-force-minimising strategies. Also, there is no current mechanistic account for the scaling of locomotion kinetics with animal size and speed. Here, we report measurements of ground reaction forces in walking children and adult humans, and their stance durations during running. We find that many aspects of gait kinetics and kinematics scale with speed and size in a manner that is consistent with minimising muscle activation required for the more demanding between mechanical work and power: spreading the duration of muscle action reduces activation requirements for power, at the cost of greater work demands. Mechanical work is relatively more demanding for larger bipeds – adult humans – accounting for their symmetrical M-shaped vertical force traces in walking, and relatively brief stance durations in running compared with smaller bipeds – children. The gaits of small children, and the greater deviation of their mechanics from work-minimising strategies, may be understood as appropriate for their scale, not merely as immature, incompletely developed and energetically sub-optimal versions of adult gaits.

## Abstract (precis)

**Highlighted Article:** The gross mechanics of walking and running children and adults support a new model for the costs dominating level terrestrial locomotion – muscle activation for mechanical work or power.

## INTRODUCTION

The benefits of adopting economical gaits are clear. However, theoretical legged gaits that minimise mechanical work require infinitely brief periods of infinitely high force and power. Take running as an example: anything other than the briefest, stiffest stance with a purely vertical force results in fore–aft accelerations and a greater demand for mechanical work to re-accelerate the body forwards. Gaits approaching the stiff-limbed work-minimising ideals may be metabolically uneconomical because there is some physiological cost to activating muscle, and muscle must be activated to provide mechanical power. At the other end of the scale, compliant gaits reduce power demands but require large degrees of leg flexion, resulting in a demand for large amounts of mechanical work. Economy may be optimised with gaits and postures that balance the muscle activation demands of mechanical power versus work. It has been recently proposed (Usherwood, 2013) that the contrasting scaling of mechanical work and power may account for the more compliant stances and relatively flexed limb posture – and generally greater deviation from work-minimising gaits – of smaller animals. This concept is developed further here to provide quantitative predictions of gait parameters, and applied to scaling of walking and running with size and speed in humans.

The aim of this paper is to identify simple but fundamental additions to work-minimising gaits to account for additional aspects of selected gait strategies, and to highlight when work minimisation is no longer the primary consideration. We compare our model predictions with measured vertical forces and stance durations in walking and running humans at a range of speeds and sizes. We take an alternative assumption to that generally found in the literature (Ivanenko et al., 2004, 2007): deviation from scaled adult walking (especially relating to ‘inverted pendulum’ mechanics) is not taken as indicating some lack of competence; rather, we assume it to be adaptive for the observed size and speed in some sense understandable from an economy perspective. Our measurements sufficiently confound age with size (and also stage of development need not be related consistently with age), that the differing influences of size and development cannot be elucidated. Instead, we consider whether characteristics of immature human gait might be understood from simple energetic issues related to scale. Specifically, we explore whether scaling accounts for children's greater deviation from work-minimising walking and running idealisations, including proportionally greater stance times and biased vertical ground reaction force traces.

**Fig. 1.**
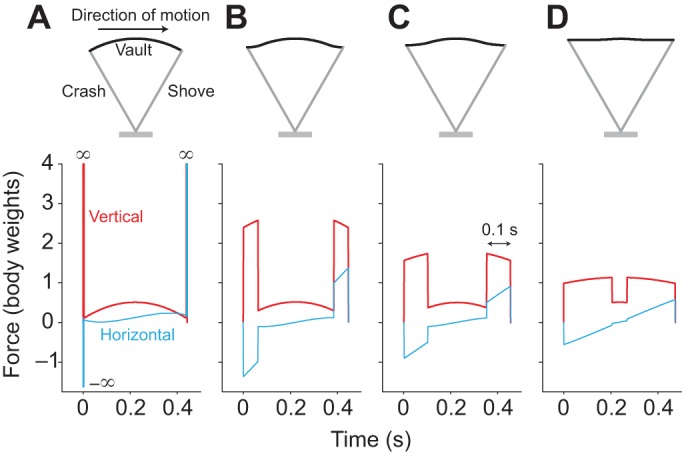
**Model development for walking.** Stance is modelled as a symmetrical, stiff-limbed inverted pendulum, with sufficient periods and magnitudes of ‘crash’ and ‘shove’ vertical (red) forces to provide weight support and horizontal (blue) to result in no net fore–aft acceleration. The work-minimising gait (A) requires infinite forces and powers; too-brief periods of muscle action (B) require excessive power; 0.1 s (C) balances work and power demands, and minimises muscle activation; too-long period (D) demands excessive work.

**Fig. 2.**
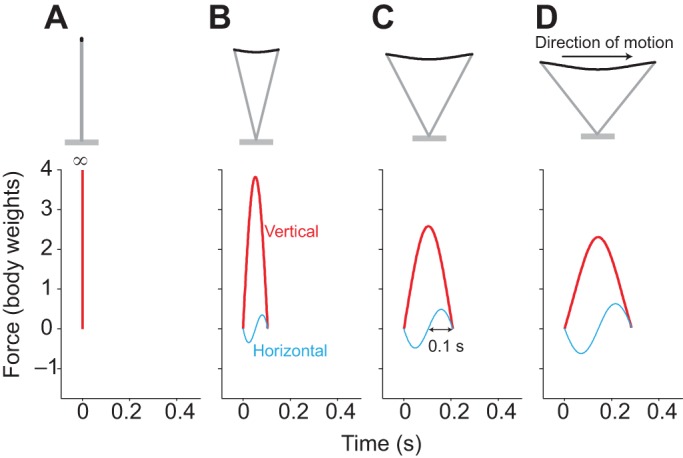
**Model development for running.** Stances are modelled for running at a range of speeds and stance periods treating the leg as a linear spring (though it is assumed that some constant proportion of the positive work demanded is due to muscle action). Impulsive stances (A: infinitely stiff, brief stance periods) minimise positive work but demand infinite power; finite but too-brief, too-stiff stances (B) demand excessive muscle activation to provide the power; intermediate stiffness (C) minimises muscle activation (resulting, at moderate speeds, in 0.1 s push-off, or a 0.2 s stance, matching work and power demands); too-compliant stances (D) result in excessive muscle activation to provide the positive work.

The gaits selected by humans vary with both speed and age or size. Simple work-minimising models of walking and running have provided considerable insight into the fundamental mechanics of locomotion of adults (Rashevsky, 1948; Alexander, 1980; Kuo, 2002; Ruina et al., 2005; Srinivasan and Ruina, 2006). In walking, the characteristic ‘M-shaped’ vertical forces experienced by each leg are broadly consistent with (though very much less extreme than) the work-minimising, impulsive, ‘inverted pendulum’ idealisation (Fig. 1A): there is a ‘crash’ at the beginning of stance; a ‘shove’ at the end of stance (though clearly neither crash or shove is actually of infinite force); and there is good quantitative agreement between observed midstance forces (the dip in the middle of the ‘M’) and those predicted as a result of the centripetal acceleration of an arcing, passively vaulting, stiff limb (Alexander, 1984; Usherwood et al., 2012). At higher speeds, impulsive running is work minimising (Rashevsky, 1948; Srinivasan and Ruina, 2006), with ballistic flight periods between each infinitely brief stance (Fig. 2A) that redirects the velocity from down to up sufficiently for the next ballistic period, providing time for each leg to be swung back ready for another stance.

**List of symbols and abbreviations**

*a*_1_, *a*_2_, *a*_3_: coefficients for sine-wave models of vertical forces in walking
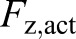
Vertical force during ‘active’ crash and shove periods of walking
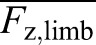
Mean vertical force acting on a leg
*F*_z,McN_: Vertical force predicted from a model combining a number of Alexander's insights
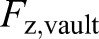
Vertical force during passive vaulting phase of walking stance
*g*: Magnitude of acceleration due to gravity (9.81 ms^−2^)
*k*_leg_: Leg stiffness
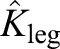
Non-dimensional leg stiffness
*L*_leg_: Leg length
*m*: Body mass
SLIP: Spring-loaded inverted pendulum
*t*: Time
*T*_stance_: Stance period
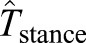
Non-dimensional stance period
*T*_step_: Step period
*V*: Speed of walking or running
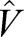
Non-dimensional speed
β: duty factor

Observed walking ground reaction forces in adult humans, while taking a form that can be broadly understood from the work-minimising impulsive inverted pendulum (Fig. 1A), clearly do not possess infinite ‘crash’ and ‘shove’ forces. Work-minimising models (without arbitrary force constraints; Srinivasan, 2010) give no account of the finite forces observed or of the increase in peak forces with increasing speed (e.g. Nilsson and Thorstensson, 1989). Furthermore, the work-minimising inverted pendulum has a symmetrical force trace about midstance; no account is made of the highly asymmetric ground reaction forces observed in small bipeds, especially children (Takegami, 1992; Diop et al., 2004; Hallemans et al., 2006; Samson et al., 2011).

Similarly, the work-minimising ‘impulsive running’ gait predicts unrealistic, infinite (though brief) vertical forces, and zero fore-aft forces. No account is given for the finite stance periods resulting in the observed finite vertical forces, and energetically relevant horizontal forces; nor how these should scale with size or speed. If work minimisation was the goal, but limb force was limited to some value, then this maximum achievable force – resulting in as near to impulsive running as possible within the limb force constraint – would be optimal at all speeds. Instead, limb forces (measured as ground reaction forces) are observed to increase with speed (e.g. Weyand et al., 2000). Despite the success of theoretical work-minimisation strategies in accounting for gross features of walking and running mechanics, including the transition between walking and running with speed, there appears to be a poor relationship between mechanical work and metabolic cost in steady, level vertebrate locomotion (see Pontzer, 2007 for a survey).

The basis of the current approach is that: (1) a cost of muscle activation dominates metabolic costs, (2) muscle has a finite work-generating capacity per contraction, (3) muscle has a finite power generating capacity during a contraction, such that (4) the extent of costly muscle activation can be attributed fundamentally to the whichever is more demanding between mechanical work and power during a contraction.

There is good empirical evidence that activated muscle volume relates closely to metabolic cost for level legged locomotion, and that metabolic cost minimisation is effective in accounting for a broad range of gait features (see Bertram and Ruina, 2001; Donelan et al., 2001). ‘Cost of force’ models have been applied to a range of animals at a range of speeds, and are highly effective at relating metabolic costs to the costs of activating muscle to impose (or oppose) forces (Taylor, 1985; Kram and Taylor, 1990; Roberts et al., 1998; Doke and Kuo, 2007; Pontzer et al., 2009). Furthermore, there is a good mechanistic account for why muscle activation might be metabolically energetic: there are considerable, measurable costs associated with simply pumping ions in and out of muscle in order to start and stop a contraction (Barclay, 2012).

The proposed model differs in that it provides an account for why muscle should be activated – what the fundamental mechanical demands are that can only be met by muscle and not some other tissue – not merely noting that its activation is metabolically costly and that activation is related to force. Previous cost-of-limb-force approaches have not provided an account for why limb forces are not reduced by extending the duration of contact, resulting in locomotion with highly compliant legs. Presumably there are work-based costs associated with longer stances, larger stance angles and greater fore–aft accelerations. Conversely, minimisation of cost of muscle force (or force rate – see Rebula and Kuo, 2015) would predict alignment of forces through joint centres, thereby making joint torques and muscle force requirements negligible [consider human standing posture or midstance posture in normal walking (Alexander, 1991; Biewener et al., 2004)]. While muscle activation due to isometric forces would indeed incur an energetic cost, simple anatomical or postural strategies – such as the heel–sole–toe walking stance in humans (Usherwood et al., 2012) – might be expected to arise for habitual, metabolically costly gaits. Postural adjustments to avoid the excess (costly) force-loading of muscles may, on occasion, be limited – accounting for the metabolic demand of holding a load on an outstretched arm, or the high metabolic cost of bipedally walking chimpanzees (Pontzer et al., 2009). However, in habitual locomotion, including walking and running in humans, posture appears to be adjustable to allow reasonably unconstrained control of mechanical advantages, changing the proportion of ground reaction forces experienced by the muscles (Biewener et al., 2004).

So, the fundamental question then becomes: why are the muscles exposed to forces during locomotion? Why are not all animal legs completely upright, with forces directed through joint centres, demanding negligible loading of the costly muscles? Here, we make the assumption that the cost of muscle activation is dominating – that the cost of performing the work per se during steady level locomotion can be neglected. However, the mechanical demands requiring muscle activation – the fundamental demand for muscles to experience loads – are assumed to be the work to be performed, and the power during, a contraction. An important departure from many previous approaches is that no ‘cost of muscle force’ is included in its own right: muscle forces are, of course, required, but we assume that only those forces that are required for the work and power demands are applied to the muscle; for habitual, steady level locomotion, we assume that anatomy and posture can be adapted to avoid any costly but non-work or power producing muscle loading.

### Premise, assumptions and model outline

We propose that the fundamental requirements for muscle loading, activation and therefore cost are: (1) mechanical work and (2) mechanical power during the contraction. The activated muscle volume for these mechanical demands are assumed to be the dominating cost for level legged locomotion, and we make and test predictions concerning features of human gaits based on the minimisation of this cost alone. Importantly, work (the positive work of the centre of mass) and power (defined here as a ‘push-off’ power, taken as the positive work over the entire duration of positive power) requirements scale with speed and size (Alexander and Jayes, 1983), and this approach can be used to make qualitative (Usherwood, 2013) and quantitative (developed here) predictions of aspects of kinetics and kinematics. We make the assumption throughout that the capacity for a given volume or mass of muscle to produce positive work and power is limited, and the ratio is: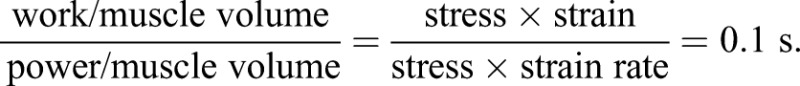 This would equate to a muscle with *V*_max_=10 lengths s^−1^ operating at high power and efficiency (0.3*V*_max_; Woledge et al., 1984) over a reasonable strain (30%), or 500 W kg^−1^ mean during contraction, and 50 J kg^−1^. While our initial 0.1 s estimate is based on a fairly extreme work and power contraction, less extreme contractions (for instance, 10 J kg^−1^ at 100 W kg^−1^, or 0.3 L s^−1^ at 3% strain) can also result in 0.1 s and leave further analysis unchanged. We should be explicit that the round value of 0.1 s is adopted here, not only because it is physiologically reasonable, but also because it provides a good fit with the kinetic and kinematic measurements without further tuning: it could be viewed as a physiologically inspired fudge-factor. We assume that this value is independent of size – this may be reasonable within a species, but is likely to be less true across species; smaller species, with higher step frequencies, may well ‘invest’ in ‘faster’ muscles, presumably at some metabolic cost (Seow and Ford, 1991). Note that this property of muscle, which we take to be fundamental to issues of balancing the costs of power and work, and predictions based on this parameter, require deviation from strict dynamic similarity, as it is dimensional (time).

While inverse and forward dynamic modelling of walking and running has provided great insight into the details of the costs associated with human gaits, they are currently constrained to considering a limited set (usually those observed) of musculoskeletal geometries. Our approach for making quantitative predictions from the simple cost function – the cost of activating a volume of muscle for whichever is more demanding between work and power – is to survey a family of gaits resulting from extensions to the point-mass work-minimising gaits (impulsive inverted-pendulum walking, Fig. 1A, and impulsive running, Fig. 2A). With this approach, we assume that an understanding of only these basic muscle properties can be informative – that geometries (‘lever arms’, ‘mechanical advantages’, ‘gear ratios’ etc.) are left as an unconsidered ‘black box’, but have been optimised through evolution of form and posture with the result of leaving the muscles exposed to such stresses, strains and strain rates as best fulfil the muscle work and power demands treated here as fundamental.

### Non-impulsive walking

We model non-impulsive walking gaits numerically as a stiff-limbed, passive (zero power) vault, and time-symmetrical periods of constant-force ‘crash’ at the start and ‘shove’ at the end of stance, with one and only one leg supporting (i.e. a duty factor of 0.5), on average, body weight over the step (and providing no net fore–aft impulse). This provides a family of gaits close to the work-minimising ideal, but allowing finite periods – and so finite forces and powers – for negative and positive work. If these finite ‘active’ periods are too brief (Fig. 1B), the work is applied over too brief a period, resulting in excess muscle activation for power. If the active periods are too long (Fig. 1D), deviation from the work-minimising gait is sufficient to result in excess muscle activation for work. Model and empirical vertical ground reaction forces are compared for walking at a range of speeds in children and adult humans.

### Non-impulsive running

**Fig. 3.**
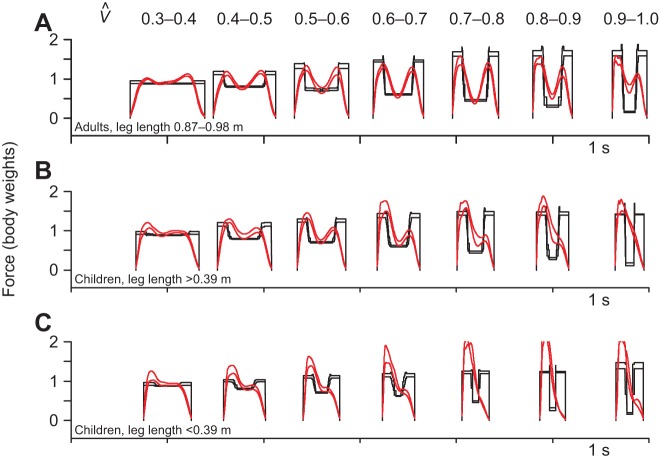
**Model and empirical vertical forces for walking at a range of speeds and sizes.** Results for an analytical approximation to the numerical walking model (black lines bounding ±1 s.d.) with 0.1 s crash and shove periods, based on empirical kinematic inputs. Measured vertical forces (red lines bounding ±1 s.d. for each group and speed bin) match well for adults, but poorly for children, especially smaller (younger) toddlers, which deviate considerably from the symmetrical inverted pendulum walking strategy. Sample sizes are shown in supplementary material Table S2.

Running gaits are modelled with spring-mass dynamics (Fig. 2) (Blickhan, 1989; McMahon and Chang, 1990; Farley et al., 1993), although the positive work is assumed to demand muscle activation. Thus, finite stance durations result in fore–aft forces, fluctuations in the fore–aft contribution to kinetic energy, and greater work requirements. Inclusion of elasticity – other than 100%, perfect elasticity, which removes any work or power demand – has no bearing on the model as it leaves the ratio of work to power unaffected. Costs for given stance parameters can be expressed in terms of required muscle volume activation (for whichever is more demanding between work and power) and is displayed normalised by the minimum for a given speed (Fig. 3). Empirical stance durations for running at a range of speeds for adults and children are presented overlying model cost contours calculated assuming constant protraction durations of 0.35 s for adults and 0.32 s for children (note the form of the contours is not highly sensitive to these values).

### Combining sine waves to report and model forces in walking

**Fig. 4.**
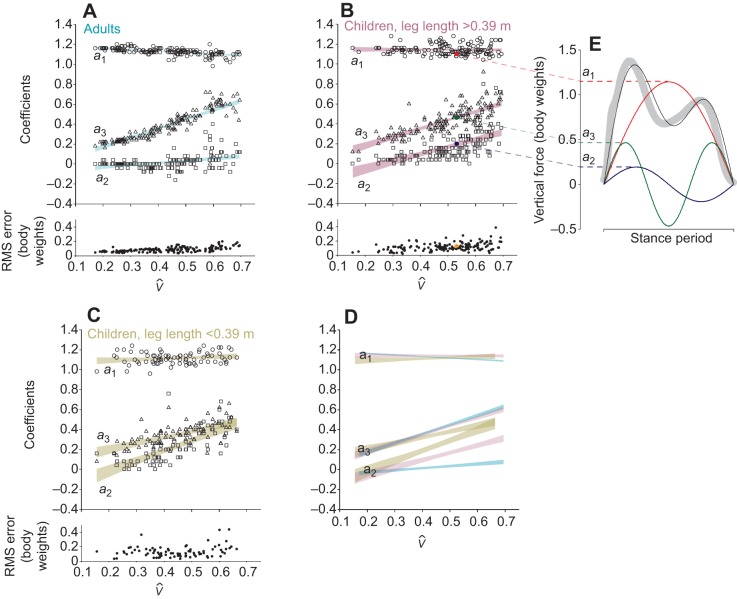
**Best-fit sine coefficients for walking vertical forces at a range of speeds and sizes.** Shown with linear regressions against dimensionless speed (±95% CI) underlying in colour for adults (blue, A), older/larger children (pink, B) and younger/smaller children (green, C), and the regressions combined for comparison (D). The coefficients relate to the amplitudes of three sine waves which, when summed, minimise the root mean square error from the measured vertical ground reaction force. The example traces (E) (black line being the reconstructed curve; underlying grey the empirical curve being fitted) relate to a specific stance denoted by coloured symbols in B. The coefficient *a*_2_ relates to a force–time bias, and increases with speed more rapidly with smaller walkers.

We use additive combinations of sine waves both to report (see Materials and methods) the relationships between walking vertical forces and speed and size, and to develop a new semi-mechanistic model for predicting walking forces. Walking force traces can be represented effectively and succinctly as three Fourier coefficients (see Fig. 4E), or amplitudes of sine waves that are added (Alexander and Jayes, 1980): *a*_1_ the amplitude of a single humped, half-sine curve through stance; *a*_2_ the amplitude of a full sine wave, with a positive value denoting a left-bias of the force-time trace; *a*_3_ the amplitude of 3/2 sine waves, summing with the first, half, sine-wave to produce the M-shaped curve.

The sine-fitting or Fourier approach of analysing force traces was originally intended as a means for reporting and comparing force traces, although it was also related to a mechanism potentially underlying changes of coefficient termed here *a*_3_ with speed (Alexander and Jayes, 1980). However, this framework does allow the development of a simple, semi-mechanistic means for predicting vertical force profiles, at least for walking adult humans. In adults, the biasing coefficient *a*_2_ is relatively small. This allows an analytical prediction for time-symmetrical vertical ground reaction forces in walking. By neglecting bias, the vertical force profile *F*_z,McN_ (to acknowledge that this model is a simple extension of a number R. McNeill Alexander's insights) through time *t* can be approximated with two coefficients *a*_1_ and *a*_3_:
(1)
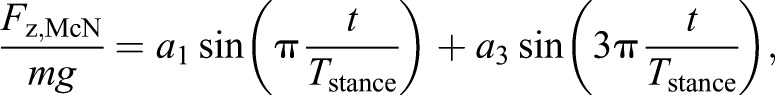
with the forces normalised by body weight. The first Alexanderesque observation (Alexander et al., 1979, originally developed to estimate peak forces in galloping) is that the vertical impulse during a stance should be sufficient to support body weight over a step of period *T*_step_. Again normalised by body weight:
(2)
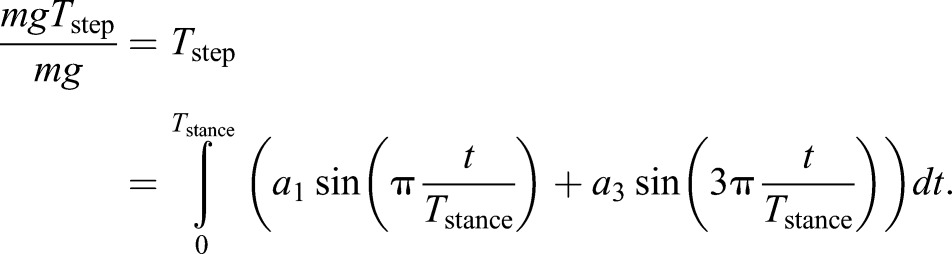
The second Alexander observation (Alexander, 1984) is that midstance forces *F*_z,vault_ in walking should be consistent with stiff-limbed vaulting about a radius of leg length *L*_leg_ at a speed *V*:
(3)
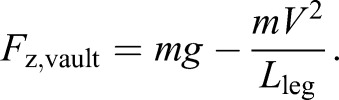
With these two independent equations, we find solutions for the two coefficients (using Mathematica, Wolfram). It is convenient here to note that:

(4)
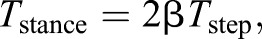
where β is the duty factor, the proportion of stride a given foot is in contact with the ground, and that speed can be related to non-dimensional speed. Throughout, speeds *V* are normalised using gravity *g* (taken as 9.81 ms^−1^) and leg length (see Alexander and Jayes, 1983 and a discussion of Froude number) to provide non-dimensional speeds 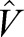:
(5)
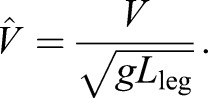
With these, the coefficients *a*_1_ and *a*_3_ required to calculate forces from Eqn 1 can be determined from easily observed non-dimensional kinematic inputs:
(6)
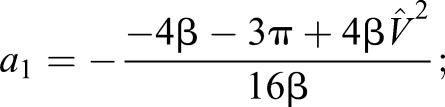

(7)
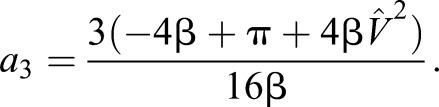


The Alexander-inspired model might best be termed semi-mechanistic: the midstance force expression, consistent with stiff-limbed vaulting, is predicted from the work-minimising inverted pendulum gait. The profile is symmetrical, also consistent with work-minimising gaits. However, the profile of the remaining forces, other than achieving net weight support, has no mechanistic basis apart from providing curves that avoid very high or rapid changes in force. Furthermore, no aspect of this approach allows any account to be made for the bias, and scaling of bias with size and speed, observed in humans.

## RESULTS

### Walking

The numerical walking model shows that all considered walking gaits with a stance duration above 0.2 s (a step frequency of below 5 Hz) would minimise muscle volume activation with an ‘active’ push-off period of 0.1 s – the work:power ratio. This observation allows a simple analytical prediction of vertical limb forces given easily observed kinematic parameters (see Materials and methods), which is applied to observed kinematics (Fig. 3) for a range of speeds and ages/sizes of humans (see supplementary material Table S1). Results for large and small children are grouped according to leg length, with a cut-off at 0.39 m. Large children (*N*=9) ranged in age from 2.5 to 4.7 years; small children (*N*=9) from 1.1 to 2.7 years.

The predicted changes in force profile broadly fit for adults up to preferred walk–run transitions speeds (
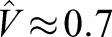
; adults were required to maintain ‘walking’ up to 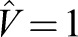): midstance forces decrease with speed, quantitatively matching stiff-limbed vaulting; peak forces increase with speed, consistent with a constant ‘active’ duration. Children were free to adopt their preferred gaits at any speed, but do not show a discrete walk–run transition. Children's force traces, especially the smaller/younger children, deviated considerably from the model predictions, showing a left bias to the force–time trace (Hallemans et al., 2006) that increased with speed.

Agreeing with previous findings for adults, *a*_3_ increased with speed, consistent with the reduction in midstance forces with speed (Alexander and Jayes, 1980). In adults, there is limited but measureable bias, increasing slightly with speed. Children, especially smaller/younger children, show a considerably greater rate of bias increase with speed (Fig. 4B–D; Table 1).

**Table 1.**
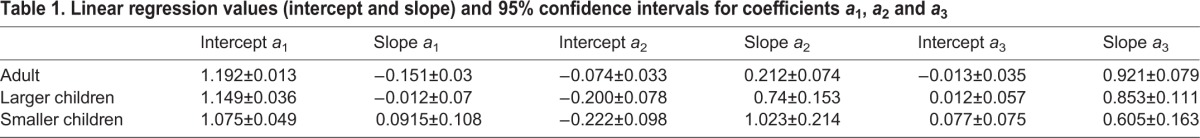
**Linear regression values (intercept and slope) and 95% confidence intervals for coefficients *a*_1_, *a*_2_ and *a*_3_**

### Running

**Fig. 5.**
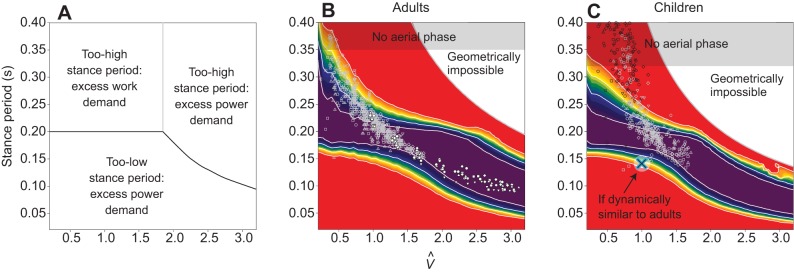
**Model and empirical results for running stance periods.** Running model results for muscle activation minimising stance durations (black line, A), cost contours and measurements (points) for adults (B) and children (C). Costs are derived for the running model of Fig. 2, for a range of speeds and step periods, calculating the activated muscle volume required to produce whichever is more demanding between mechanical work or power. Cost contours are presented normalised by the minimum value (of activated muscle volume) for each speed, with white contours indicating 5% boundaries above minimal (purple); red regions indicating greater than 20% above minimal required activation. Points denote empirical observations for undergraduates (grey points, B) and near-elite sprinters (white points, B; data from McGowan et al., 2012), and children with duty factor above 0.5 (black points, C) and below 0.5 (grey points, C). At moderate speeds, a stance period of 0.2 s is predicted to be optimal independent of leg length – for both adults and children – and this is close to empirical observation for running adults and children; at 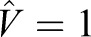, the current model provides a much better prediction for children than simple dynamic similarity, which would suggest (blue outline cross) much briefer stances. High stance periods at high speeds are geometrically impossible if stance length exceeds double leg length; stance periods greater than swing result in no aerial phase (duty factor >0.5).

Up to moderate running speeds, the model conditions for minimising activated muscle volume match those for walking: this cost is minimised when work and power demands are equal, with a positive power duration of 0.1 s. Given a symmetrical stance, this equates to a stance duration of 0.2 s, independent of size. Stance durations shorter than this require excess muscle activation because of a too-brief active period and high power demand (some work is always demanded, even with stiff legs and brief stances, because of the vertical motions imposed by running with finite protraction periods). Stance durations longer than this require excess muscle activation as a result of high work demands because of fore–aft forces and velocity fluctuations. The model indicates (Fig. 5A) that, at higher speeds, power demands for muscle activation dominate, and briefer stances reduce power due to reducing work (despite also reducing the period of activation).

Empirical measurements (points, Fig. 5B,C) do not fit the model optimum stance periods precisely. At low speeds, observed stance durations are considerably higher than predicted; however, this deviation is in a relatively flat region of the cost surface. Given the extreme simplicity of the model, the match appears good at normal running speeds (
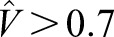
), accounting for the reduction in stance duration observed in sprinting. Furthermore, the model provides an effective account for the similarity in absolute stance periods (approximately 0.2 s) at comparable non-dimensional speeds (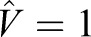) for adults and children, despite considerable differences in leg length. Were the gaits to follow dimensional similarity, stance period *T*_stance_ would depend on leg length (Alexander and Jayes, 1983):
(8)
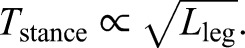
Thus, if an adult of *L*_leg_=0.93 m at 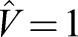 has a stance period of 0.2 s, a child of *L*_leg_=0.46 m following dynamic similarity would have *T*_stance_=0.14 s; instead, values much closer to 0.2 s are observed (blue cross, Fig. 5C).

## DISCUSSION

### Walking

**Fig. 6.**
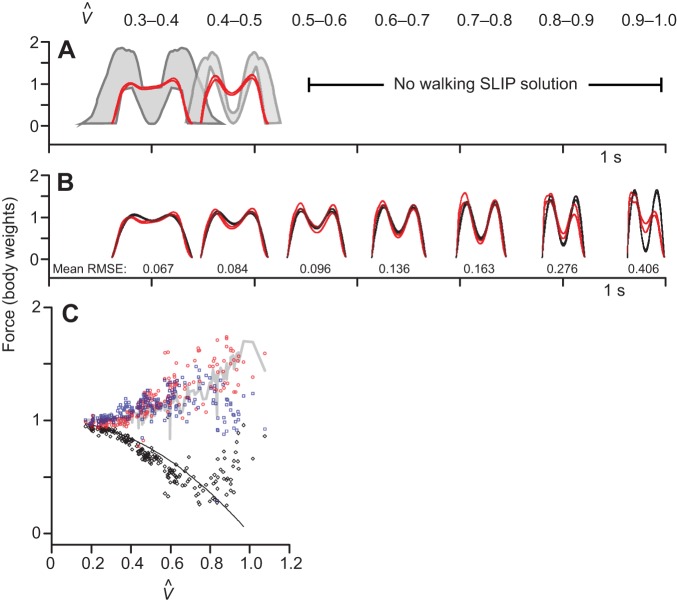
**Results for alternative reductionist accounts for the vertical forces of bipedal walking.** Underlying grey regions (A) denote the range of possible walking-like outcomes (symmetrical, with a broadly ‘M’-shaped profile) for the linear spring-mass model (or spring-loaded inverted pendulum or SLIP model) with appropriately tuned parameters. At low speeds, realistic forces can be found; at medium speeds, midstance forces are under-predicted; and no walking solutions can be found at high walking speeds (following Geyer et al., 2006). A simple, semi-mechanistic analytical model developed here on the assumptions and principles of Alexander provides remarkably good fits given only observed speed, leg length and duty factor (black lines, B, show ±1 s.d. using observed kinematic inputs; red lines show ±1 s.d. of empirical data for adults for each non-dimensional speed bin). Model midstance forces (black line, C) agree with measured minimum forces in the trough of the M and modelled maximum forces (grey line) agree with measured first peak (red points) and second peak (blue points), at least up to preferred walk-run transitions speeds (
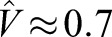
). However, the ‘Alexanderesque’ approach has limited mechanistic basis, and does not provide an account for why peak forces increase with walking speed.

Before exploring the implications of the proposed mechanism underlying aspects of gait selection, it is helpful to highlight contrasts with some other general approaches for understanding the basic mechanics of bipedal gaits. Firstly, spring-mass models, including ‘spring-loaded inverted pendulum’ or SLIP models, offer an appealing, apparently mechanistic framework for considering both walking and running. Remarkably, linear spring parameters can be found that not only demonstrate running-like ‘bouncing’ gaits with a single, approximately half-sinusoidal vertical ground reaction force, but the same leg properties (given appropriate initial conditions) can also produce walking-like gaits with M-shaped vertical force traces which, at low speeds, provide a good match with empirical force measurements (Geyer et al., 2006; Fig. 6). However, at moderate walking speeds the match becomes poor, with all observed walking-like solutions showing midstance forces considerably below those predicted (Fig. 6). Furthermore, no stable walking-like gaits can be found at higher walking speeds (Geyer et al., 2006), limiting the predictive value of the SLIP model for walking.

When *a*_1_ and *a*_3_ are applied to Eqn 1 using empirical duty factor, speed and leg length, the Alexanderesque approach provides an excellent fit with observed vertical forces in walking adults (Fig. 6B,C), at least up to the preferred walk–run transition speed. However, this approach is only semi-mechanistic, providing no reasoning behind why – other than as a mathematical outcome of combining sine waves – peak forces should increase with speed.

The approach adopted in this paper seeks to explore whether a simple but fundamental physiological cost might underlie deviations from work-minimising walking and running. In walking adults, this approach appears broadly successful in accounting for the magnitude of peak and trough forces, and their scaling with speed. An ‘active’ duration of 0.1 s, given reasonable muscle power and work properties, minimises the volume of muscle to be activated for whichever is more costly between work and power. While the activation-minimising model cannot produce the elegant curves matching the adult force data as generated by the Alexanderesque-approach, it does provide a mechanistic account for why peak forces increase with speed: muscle activation is minimised if the duration of mechanical power is 0.1 s – so a reduction in midstance force with speed demands an increase in peak forces if net weight support is to be achieved.

### Running

**Fig. 7.**
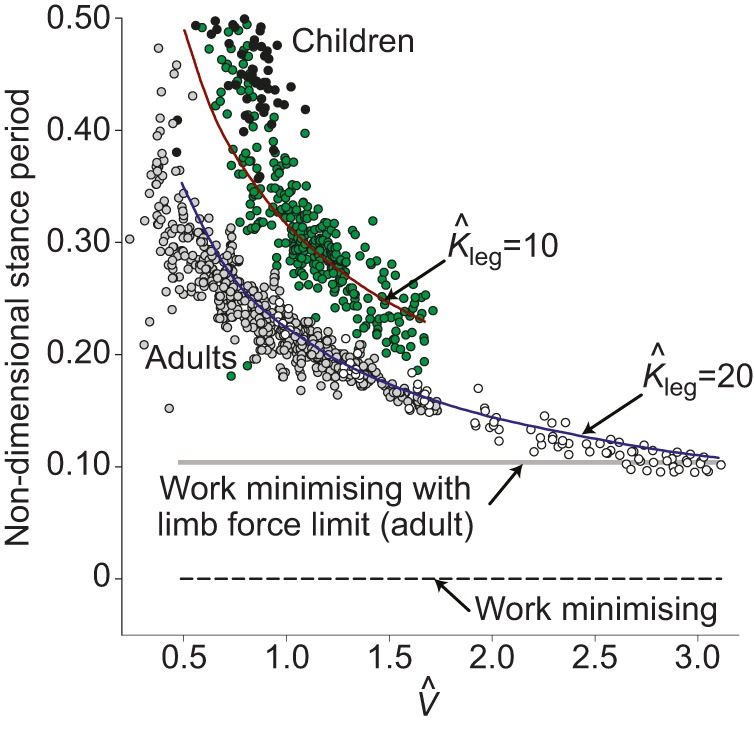
**Results for alternative accounts for stance durations in running.** Empirical stance durations presented in non-dimensional form for running undergraduates (grey), sprinters (white), children (green) and children with a duty factor greater than 0.5 (black). Children are not dynamically similar to adults: their stance durations are disproportionately high. Stance durations found from spring-mass models with appropriate leg length, stance duration and non-dimensional leg stiffness 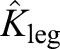 provide a good match for adults (blue line) and children (red line). However, spring-mass models do not have a mechanistic basis, do not account for why leg stiffness should stay approximately constant across speed, and provide no account for the relatively more compliant legs of children. Theoretical mechanical work minimising running requires infinitely small stance durations (dashed lines) and infinite forces. Work-minimising gaits with a constrained maximum limb force would result in constant, minimal stance duration (grey line for adults), failing to account for higher stance durations at lower speeds.

Running mechanics is often represented as some form of spring-mass system (Blickhan, 1989; McMahon and Chang, 1990; Farley et al., 1993). When appropriate parameters are tuned for steady running for adults [using *L*_leg_=0.933 m, a protraction period of 0.35 s, and a non-dimensional leg stiffness 
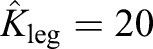
, where 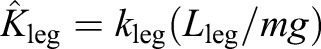 following McGowan et al., 2012] across a range of speeds, stance durations can be found. The model lines and data are shown in Fig. 7, using a non-dimensional form of 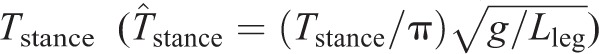. The fit is clearly good; however, there no underlying mechanism accounting for why that leg stiffness should be selected, or why it should remain approximately constant (if slightly stiffening with speed; McGowan et al., 2012); the muscles and tendons of a biological leg certainly do not constitute an obligate spring of constant stiffness. Furthermore, no account is made of the relatively more compliant legs of children (model line for 
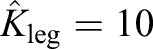
 found taking *L*_leg_=46 m and a protraction period of 0.32 s). The presentation of stance period in non-dimensional form highlights the deviation from dynamic similarity: children show disproportionately long stance durations.

As with walking, stance periods for running at running speeds broadly match the predictions from activated muscle volume minimisation, both across speed (leading to a stance duration of 0.2 s up to moderate speeds, decreasing at higher sprinting speeds) and size (absolutely similar stance durations in children – at the same non-dimensional speed – as adults). There appear to be no competing models with a clear mechanistic basis that would account for the general observations of scaling with speed and size in running. However, measured stance durations are considerably above the predicted 0.2 s at low non-dimensional speeds, especially for children. While this deviation does occur at the relatively shallow region of the cost surface (a similar deviation but towards too-brief stance periods would be predicted to be very much more costly), the current model does fail in this aspect. It should be noted that gait mechanics at non-dimensional speeds between 0.7 and 1.0 do not fit neatly into discrete ‘walking’ and ‘running’ gaits for children (Fig. 3); and theoretical work-minimising gaits (Srinivasan and Ruina, 2006) and observed bird gaits (Usherwood, 2010) do not fit within traditional walking and running paradigms.

### Alternative accounts

The differential scaling of work and power with size may account not only qualitatively for the scaling of posture (Usherwood, 2013), but quantitatively for the scaling of stance parameters in running: short-legged runners require relatively longer stance durations – higher duty factors – because of the disproportionately higher power demands at smaller scales. This may explain why small birds (Gatesy and Biewener, 1991) and children show a blurred walk–run transition, ‘running’ with marginal or no aerial phase and deviating from dynamic similarity (*sensu* Alexander, 1977; Alexander and Jayes, 1983) with larger bipeds. Furthermore, the model provides a means for understanding the energetically costly deceleration phase of stance in running from fundamental muscle properties: for a steady run, this is required in order to ‘buy’ time over which work can be applied, reducing the muscle activation required for power. This time leads to costly fore–aft forces given: (1) feet are not on skates or wheels, and cannot travel along the ground, and (2) torques about the centre of mass are costly, even though they do reduce fore–aft forces to a measureable extent that is broadly consistent with work minimisation (Usherwood and Hubel, 2012).

Alternative suggestions for why animals do not always use more stiff-limbed, upright gaits, perhaps especially so when small (Biewener, 1989; Gatesy and Biewener, 1991), cannot be discounted. Clearly, infinitely brief stances and completely stiff running legs, while offering theoretical mechanical work minimisation, would impose catastrophically damaging loading. But if some value of limb force can be withstood, why is the minimum stance duration consistent with this maximum force – resulting in minimum work demand within the force constraint – not selected at all speeds (Fig. 7)? This could be because of some scaling in Safety Factor with speed, allowing more risky stances at higher speeds. In addition, animals might benefit from a crouched posture and finite stance duration to provide the potential for acceleration, manoeuvrability or climbing each step. It is not clear, however, why these advantages would benefit smaller animals (especially considering their higher step frequencies) disproportionally. Such issues are reasonable and cannot be discounted with the approach presented here. However, simple minimisation of muscle activation does provide a parsimonious and broadly quantitative account for scaling of a range of walking and running kinematics and kinetics.

### Accounting for bias: semi-impulsive walking

A notable failing of the approach introduced so far in this paper is the presence and scaling of biased force–time traces in walking, particularly among small children. One account for this is that, at smaller scales, a key initial premise is incorrect: activated muscle volume would be minimised with a gait deviating only slightly from the time-symmetrical, work-minimising gaits. Shorter-legged walkers are predicted to deviate more from the impulsive, work-minimising gaits with longer relative durations of ‘active’ pushing (see Fig. 3C – small, fast-walking children would be predicted to have only very brief vaulting periods). Given that simple scaling arguments indicate that smaller animals may be relatively more influenced by issues relating to power, might the asymmetric forces be considered a strategy for ameliorating power demands? In order to explore this, we develop a model for an extreme form of the biased walking strategy, which we term ‘semi-impulsive walking’.

**Fig. 8.**
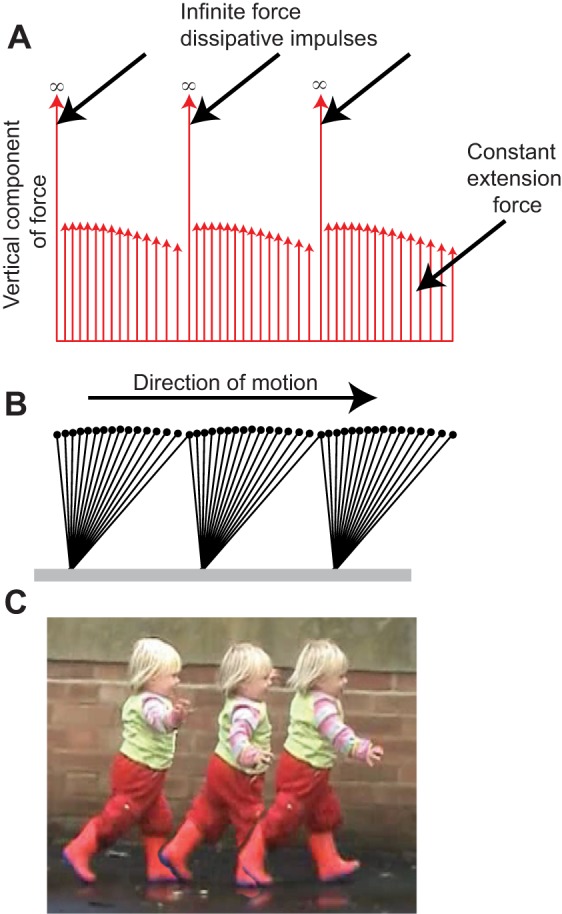
**Vertical forces and stick-figure kinematics for three exemplar steps of the semi-impulsive walking model.** (A,B) With short legs, small bipeds can reduce the muscle activation demands by reducing power through extending the leg extension phase throughout stance, despite greater deviation from the symmetrical, work-minimising inverted pendulum gait (Fig. 1). Semi-impulsive walking results in an asymmetric kinematic and force profile, with a relatively upright, high-force early stance, and an extended, inclined leg at the end of stance; qualitatively similar to the forces measured for children (Figs 3 and 4) and easily identified in toddlers (C).

This development assumes that the costs of negative (dissipative) work and power are small (negligible) compared with positive work and power; the ‘crash’ is treated as impulsive, occurring over a very brief period. This allows a family of semi-impulsive walking gaits to be modelled in which a constant force extends the leg, spreading positive work application throughout stance, with the work demands calculated from a stiff, plastic collision (see Kuo, 2002 or Ruina et al., 2005) crash at the beginning of the next stance. Assuming exactly one leg supports the body at any time, and numerically optimising the magnitude of the constant extension force so as to avoid net fore–aft acceleration while providing net weight support, gaits can be found for given speeds and step lengths. These are characterised by a relatively vertical, short leg in early stance with an early-stance vertical force bias (Fig. 8), and an extending leg throughout stance finishing with a relatively extended, inclined leg at the end of stance, before the dissipative ‘crash’ at the beginning of the next step. Something close to this strategy is clearly visible (sometimes even audible) in toddlers (Fig. 1C and Fig. 8C), and is predicted through the scaling of activated muscle volume minimisation. While we model two walking extremes (non-impulsive and semi-impulsive), we do not attempt to survey the entire parameter space between the two. Although we can calculate a predicted transition between the two gaits (semi-impulsive is predicted at smaller sizes and shorter steps), this should not be treated as a quantitative prediction for asymmetry; the transition is likely to be graded. However, the revealed principle appears reasonable: bipeds with briefer steps benefit from reducing the muscle activation costs due to power by applying work throughout the majority of stance, despite greater deviation from work-minimising, symmetrical, impulsive inverted-pendulum walking. This provides a contrasting, but not necessarily conflicting, account for asymmetrical ground reaction forces from recently proposed models for birds (Andrada et al., 2014; Birn-Jeffery et al., 2014), human running (Maykranz and Seyfarth, 2014) and sprinting (Clark et al., 2014).

### Conclusion

The minimisation of muscle volume activated for whichever is more demanding between mechanical work and power successfully provides a simple, general and mechanistic account for features of walking and running mechanics, and their scaling with speed and size in humans. Aspects of small children's gaits – higher duty factor, more biased walking forces and greater deviation from work-minimising gaits than adults – have similarities with those of medium-sized birds, and may be related to adaptive strategies for limiting the muscle activation demands due to power.

## MATERIALS AND METHODS

### Empirical measurements

Eighteen children ranging in age from 1.1 to 4.7 years, and leg length (from ground to greater trochanter during standing) of 0.31 to 0.525 m, and five adults (leg length from 0.87 m to 0.98 m) locomoted at a range of speeds over a 4.8 m by 0.9 m array of eight forceplates (at 500 Hz; Kistler 9287B). Measurements were approved by The Royal Veterinary College ethics committee, and were performed after informed consent or parental consent. Children were free to select their preferred gaits; adults were also required to extend their walking above their preferred walk–run transition speed (
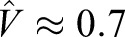
). Younger children were closely accompanied by their parents, and often varied speeds; no measurements are included where contact was made with a parent, and any step with a change of greater than 0.2 m s^−1^ was excluded. Anonymised ground reaction force data are available as supplementary material Table S1. Vertical limb forces are presented (Fig. 3) divided according to speed and leg length, resulting in uneven sample numbers, whether in terms of subject or trials (supplementary material Table S2).

Footfall timing parameters were measured using optical motion capture (250 Hz, Qualisys, Gothenbury, Sweden) of a toe marker for seven adults running at a gradually ramped range of speeds on a treadmill. Running kinematic data are supplemented (Fig. 5C) by values for 12 highly competent, specialist sprinters (McGowan et al., 2012).

### Derivation of analytical approximation for vertical limb forces in walking from simple kinematic inputs

This model is based on the requirements of mean vertical weight support due to a stance composed of two ‘active’ periods (the ‘crash’ and the ‘shove’), of period *T*_act_ and force *F*_act_, at each end of one passive, stiff-limbed vaulting period of force *F*_z,vault_ over period *T*_vault_. The model does not approach variation of forces within each period; the predicted force profile is a blocky, symmetrical ‘M’ shape (Fig. 3).

The impulse from a leg over the entire stance (of the single leg) is the same as that for the whole body over a step, and equals the sum of the impulses from both ‘active’ periods and the vaulting period:
(9)
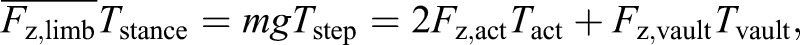
where 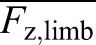 is the mean vertical force experienced by the limb over the stance period. The vaulting period is given by:
(10)
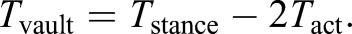
The vertical forces during the vaulting phase can be effectively modelled given the centripetal acceleration of the body mass arcing about the foot – Eqn 3. Thus, if we have a value for *T*_act_ found from the numerical model to minimise muscle activation (that which makes work and power demands equal, found to be the work:power ratio, or 0.1 s), we can combine Eqns 9 and 10 to find the force during the ‘active’ periods at beginning and end of stance:
(11)
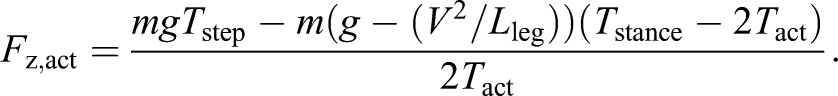
With this, gross aspects of walking vertical force profiles can be predicted from body weight and easily observed kinematic inputs (Fig. 3), from the mechanistic principle of a work-minimising strategy with small adjustments to reduce the activation costs due to power demands.

### Fitting of three sine amplitudes to vertical force data

Best-fit coefficients for vertical force traces were found for curves of the form
(12)
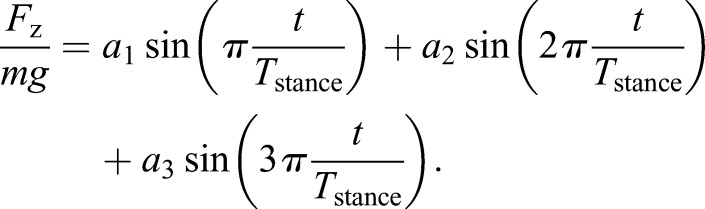
See Fig. 4E for a graphical representation of these mathematics. Best fit coefficients *a*_1_, *a*_2_ (a positive value indicating an early bias in force) and *a*_3_ for each force trace were determined by finding the global minimum of square root mean error between observation and model using increments of 0.02 body weight. Results for adults, large/old children and small/young children are shown in Fig. 4; linear regression parameters for the dependence on non-dimensional speed in Table 1.

## Supplementary Material

Supplementary information
