# Supplementary material for: Children and adults minimise activated muscle volume by selecting gait parameters that balance gross mechanical power and work demands
Source: J Exp Biol. 2015 Sep;218(18):2830–9. doi: 10.1242/jeb.122135 (PMC4582168; doi:10.1242/jeb.122135)
Supplement: Supplementary information [file supp_218_18_2830__index.html]

Supplementary information 

# Children and adults minimise activated muscle volume by selecting gait parameters that balance gross mechanical power and work demands

## JEB122135 Supplementary information

- Supplementary information
