# Supplementary material for: Children and adults minimise activated muscle volume by selecting gait parameters that balance gross mechanical power and work demands
Source: J Exp Biol. 2015 Sep;218(18):2830–9. doi: 10.1242/jeb.122135 (PMC4582168; doi:10.1242/jeb.122135)
Supplement: Supplementary information [file supp_218.18.2830_JEB122135supp.pdf]

Table S1.

Click here to Download Table S1

Table S2. Sample sizes for data shown in Figs 3 and 4.

	$\hat{V}$:	0.3-0.4	0.4-0.5	0.5-0.6	0.6-0.7	0.7-0.8	0.8-0.9	0.9-1.0
Adult subjects		3	5	5	5	4	5	3
Adult walking trials		33	39	32	22	10	19	12
Large child subjects		8	7	6	6	5	4	3
Large child trials		22	36	41	35	18	22	6
Small child subjects		8	6	7	5	2	1	1
Small child trials		21	23	15	11	2	2	2

Large and small children are separated by having leg lengths longer or shorter than 0.39 m. Large children (*N*=9) ranged in age from 2.5 to 4.7 years; small children (*N*=9) from 1.1 to 2.7 years.

Journal of Experimental Biology • Supplementary information
